# High stroma‐derived WNT5A is an indicator for low‐risk prostate cancer

**DOI:** 10.1002/2211-5463.13131

**Published:** 2021-03-11

**Authors:** Wadim Kisel, Stefanie Conrad, Angelika Borkowetz, Giulia Furesi, Susanne Füssel, Ulrich Sommer, Martina Rauner, Christian Thomas, Gustavo B. Baretton, Klaus‐Dieter Schaser, Christine Hofbauer, Lorenz C. Hofbauer

**Affiliations:** ^1^ University Center for Traumatology, Orthopedics and Plastic Surgery Technische Universität Dresden Germany; ^2^ Division of Endocrinology, Diabetes, and Bone Diseases Department of Medicine III and University Center for Healthy Aging Technische Universität Dresden Germany; ^3^ Department of Urology Technische Universität Dresden Germany; ^4^ Department of Pathology Technische Universität Dresden Germany; ^5^ University Cancer Center Technische Universität Dresden Germany

**Keywords:** prostate cancer, risk, stromal tissue, WNT5A

## Abstract

Prostate cancer (PCa) is a major cause of cancer‐related death in men. Tumor‐derived protein derived from Wnt5A gene (WNT5A) plays an important role in primary and metastatic PCa. Surrounding stroma cells also produce WNT5A, which may modulate the biology of PCa. Here, we assessed the role of stroma‐derived WNT5A (stWNT5A) in primary PCa. A tissue microarray of samples obtained from 400 patients who underwent radical prostatectomy and control samples from 41 patients with benign prostate hyperplasia (BPH) was immunohistochemically assessed for expression of stWNT5A. The cores were scored for staining intensity: 0 (no staining), 1 (weak), 2 (moderate), or 3 (strong) and the stained stromal surface area: 0 (0%), 1 (1–25%), 2 (26–50%), 3 (51–75%), or 4 (76–100%). Gleason Score (GS) and TNM‐stage were assessed by stratifying the cohort into high‐risk (≥ pT3, pN1, GS ≥ 8) and non‐high‐risk patients. Ki67 and TUNEL assays were performed to assess proliferation and apoptosis. Expression of stWNT5A in BPH and tumor‐free control samples was 1.2‐fold higher compared to tumor samples (*P* < 0.001). Non‐high‐risk patients had a higher stWNT5A score than high‐risk patients (*P* < 0.05). stWNT5A expression was not correlated with overall and cancer‐specific survival. Proliferation (*r* ^2^ = 0.038, *P* < 0.001) and apoptosis (*r* ^2^ = 0.277, *P* < 0.001) negatively correlated with stWNT5A expression. In summary, we show that expression of stWNT5A is higher in benign tissue and non‐high‐risk PCa. Stroma‐derived Wnt signaling and tumor‐derived Wnt may differentially impact on tumor behavior. Future studies are warranted to dissect the Wnt profile in tumor vs. surrounding stroma tissues.

## Graphical abstract

Protein derived from Wnt5A gene (WNT5A) plays an important role in primary and metastatic prostate cancer (PCa). Tumor cells and surrounding stroma cells produce WNT5A. In a tissue microarray, we assessed the role of stroma‐derived WNT5A (stWNT5A) in primary PCa. Expression of stWNT5A is higher in benign tissue and non‐high‐risk PCa.

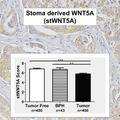

**Abbreviations**
BPH: benign prostate hyperplasia
CSS: cancer‐specific survival
FZD5: Frizzled 5
GS: Gleason Score
IQR: interquartile range
Ki67: Kiel‐67
OS: overall survival
PCa: prostate cancer
PSA: prostate‐specific antigen
ROR2: Tyrosine‐protein kinase transmembrane receptor
RP: radical prostatectomy
RYK ICD: nuclear receptor tyrosine kinase
RYK: cytoplasmatic receptor tyrosine kinase
stWNT5A: stroma‐derived WNT5A
TMA: tissue microarray
TNM‐: staging classification for tumor diseases, regarding local tumor spreading (T), lymph node metastases (N) and distant metastases (M)
TUNEL: TdT‐mediated dUTP‐biotin nick end labeling
Wnt: portmanteau of ‘Wingless’ and ‘Int‐1’
WNT5A: protein derived from Wnt5A gene

Prostate cancer (PCa) is the most common malignant tumor [1] and the third leading cause of cancer‐related death for men in Europe [2]. In advanced disease, many patients develop bone metastases [3]. At this stage, PCa is uncurable. Bone serves as a reservoir for dormant tumor cells that resist chemotherapy and release metastatic cells to other tissues [4]. With current therapies in patients with metastatic disease, the 5‐year survival rate is only 30% [5].

In physiological conditions, portmanteau of ‘Wingless’ and ‘Int‐1’ (Wnt) signaling plays a key role in embryonic development [6, 7] and regulation of cell proliferation, cell polarity, differentiation, migration, communication, and cell survival. Two pathways exist the canonical, β‐catenin‐dependent pathway [8, 9] and the noncanonical, β‐catenin‐independent pathway [10]. The highly evolutionary conserved noncanonical Wnt pathway is important in planar cell polarity, convergent extension, and epithelial‐mesenchymal interaction [11]. Depending on cancer entity, tumor‐promoting [12, 13, 14, 15] and tumor‐suppressive effects [16, 17, 18, 19, 20] have been reported.

Studies in PCa have revealed an ambivalent role of tumor‐derived WNT5A. Some investigations show its association with increased aggressiveness [15, 21], whereas others revealed a better outcome of PCa patients after radical prostatectomy (RP) highly expressing WNT5A [22, 23]. In addition, WNT5A is also known for its role to maintain bone homeostasis [24] and to control the dormancy of PCa cells [25] in the bone microenvironment.

Our previous findings suggested that high expression of WNT5A in primary PCa tissue is associated with longer overall survival (OS), but not cancer‐specific survival (CSS). Furthermore, overexpression of WNT5A increased apoptosis and decreased proliferation of PCa cells *in vitro* and reduced tumor burden *in vivo* [26]. The cancer‐surrounding stromal tissue has recently become the subject of research as a potential regulative factor. A study of prostate gland physiology in normal mice demonstrated a suppressive role of stroma tissue‐derived WNT5A on proliferation of epithelial cells [27]. Furthermore, mesenchymal stroma cells were involved in the development of metastases in the bone [28]. As WNT5A can act as a chemoattractant for PCa cells [29], we suppose that stromal tissue‐derived WNT5A (stWNT5A) may be associated with the outcome of PCa patients. Therefore, the goal of this study was to investigate the role of stWNT5A in primary PCa.

## Results

### Expression of stWNT5A in tumor is lower than in tumor‐free and BPH tissues, but shows no association with survival

A tissue microarray (TMA) of 400 men was analyzed regarding expression of stWNT5A. Characteristics of the cohort are shown in Table 1. This cohort was enriched with 81.25% (*n* = 325) high‐risk patients, and hence, it did not represent a normal cohort of men with primary PCa. During a median follow‐up‐period of 11.2 years [interquartile range (IQR) 9.2–13 years], a total of 111 (27.8%) patients died of any cause or PCa, of which 38 patients because of PCa.

**Table 1:** Characteristics of the cohort. TNM, pathological classification of malignant tumors regarding local tumor spreading (pT), lymph node metastases (pN), and distant metastases (M).

Characteristics of the cohort	Rate
Median age at diagnosis in years (IQR)	62 (61, 68)
Median time to death of any cause in years	8 (4.74, 9.69)
Preoperative median PSA (IQR) in ng·mL^−1^	8.6 (5.54,15.58)
TNM‐stage, frequency (%)	
pT2	178 (44.5)
pT3	154 (38.5)
pT4	68 (17)
pN0	294 (73.5)
pN1	106 (26.5)
Postoperative GS, frequency (%)	
≤ 6	78 (19.5)
=7	75 (18.75)
≥ 8	247 (61.75)
Clinical risk, frequency (%)	
Non‐high‐risk patients (≤ pT2 and/or pN0 and/or GS ≤ 7)	75 (18.75)
High‐risk patients (≥ pT3 and/or pN1 and/or GS ≥ 8)	325 (81.25)

**Fig. 1:**
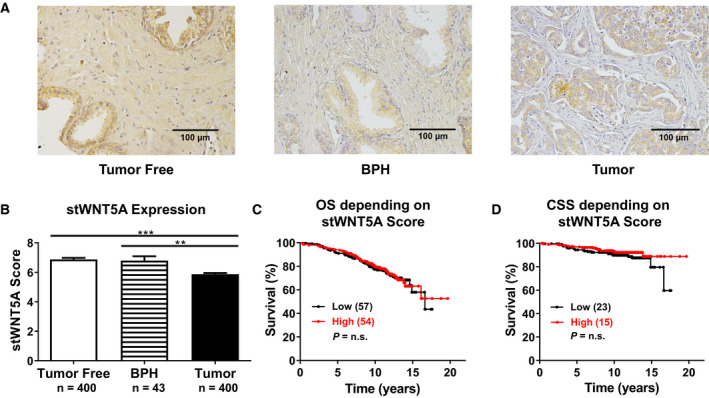
Expression of stromal WNT5A (stWNT5A) is lower in PCa compared to tumor‐free tissue and BPH, but shows no correlation to survival. (A) Representative immunohistochemical staining of stWNT5A in tumor‐free tissue, BPH and PCa tumor tissue. (B) Scoring of intensity (0–3) and quantity (0–4) of stWNT5A in tumor‐free tissue (*n* = 400), BPH (*n* = 43), and PCa tumor tissue (*n* = 400). (C‐D) The cohort was divided at the median of the stWNT5A score (5.875) in a low‐expression (Low, *n* = 200) and a high expression group (High, *n* = 200). The graphs show C OS and D CSS curves of these groups (dead patients in brackets). Scale bar equates to 100 µm. Normal distribution was declined by Kolmogorov–Smirnov test (*P* < 0.05) for B, here Mann–Whitney *U*‐test was conducted for single group comparisons. Differences in survival were calculated using Log‐Rank (Mantel‐Cox) test. Data are shown as mean ± SD or survival curves, ***P < 0.001, **P < 0.01.

The immunohistochemical staining revealed a different intensity and quantity pattern of stWNT5A in tumor compared to tumor‐free or benign prostate hyperplasia (BPH) tissue (Fig. 1A). In tumor tissue, stWNT5A expression was significantly lower than in tumor‐free and BPH tissue (Fig. 1B, *P* < 0.001). The cohort was dichotomized at the median of the stWNT5A score (5.875) into a low‐expression and a high expression group. The comparison of patients with low stWNT5A and high stWNT5A has shown no significant difference for OS (Fig. 1C, *P* = 0.09) and CSS (Fig. 1D, *P* = 0.09).

### High stWNT5A goes along with low‐risk PCa, thus with a lower Gleason Score and localized PCa without lymph node metastases

The cohort was analyzed for the relevant parameters [30] serum prostate‐specific antigen (PSA), GS, and TNM‐stage and divided according to clinical risk stratification guidelines for PCa for local recurrence and development of metastases after prostatectomy [31]. As prostatectomy had already been performed, low‐ and intermediate‐risk patients were summarized to a non‐high‐risk (≤ pT2 and pN0 and GS ≤ 7) and a high‐risk group (≥ pT3 and/or pN1 and/or GS ≥ 8).

**Fig. 2:**
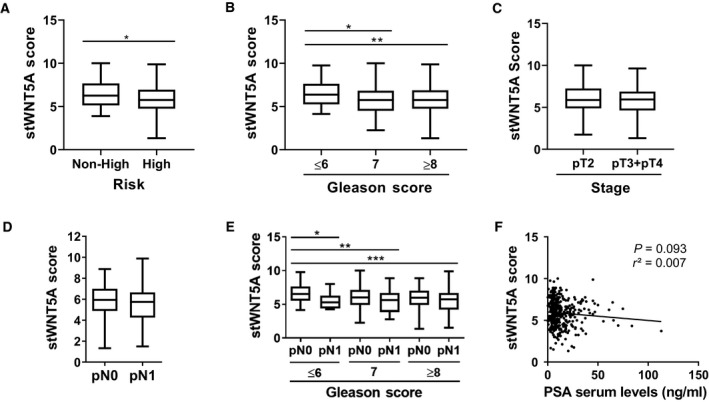
Non‐high‐risk PCa is associated with high expression of stWNT5A, thus with a lower GS and localized PCa without lymph node metastases. (A) Comparison of stWNT5A expression in non‐high‐risk (< pT3, pN0, GS < 8, *n* = 75) and high‐risk patients (≥ pT3 and/or pN1 and/or GS ≥ 8, *n* = 325). (B) Comparison of stWNT5A expression in patients with GS ≤6 (*n* = 78), GS = 7 (*n* = 75), and GS ≥ 8 (*n* = 247). (C‐D) Regarding the TNM classification, stWNT5A expression was analyzed for local spreading and absence or presence of lymph node metastases. (C) Expression of stWNT5A in localized (pT2, *n* = 178) and locally advanced (pT3 and pT4, *n* = 222) PCa. (D) Expression of stWNT5A in pN0 (*n* = 294) and pN1 (*n* = 106) patients. (E) The cohort was divided into GS ≤ 6, GS = 7, and GS ≥ 8. These groups were examined for the expression of stWNT5A in pN0 and pN1 patients (pN0/GS ≤ 6 *n* = 67, pN1/GS ≤ 6 *n* = 11, pN0/GS = 7 *n* = 37, pN1/GS = 7 *n* = 38, pN0/GS ≥ 8 *n* = 190, pN1/GS ≥ 8 *n* = 57). (F) Linear regression analysis of stWNT5A to PSA. Student’s *t*‐test was conducted for single group comparisons in B‐E. Normal distribution was declined by Kolmogorov–Smirnov test for A (*P* < 0.05); here, Mann–Whitney *U*‐test was conducted for group comparison. Data are shown as box plots. Correlation was calculated using Pearson *r* correlation and linear regression analysis, *P < 0.05, **P < 0.01, ***P < 0.001.

Non‐high‐risk patients displayed a significantly higher expression of stWNT5A compared to high‐risk patients (Fig. 2A, *P* < 0.05). However, OS (*P* = 0.125) and CSS (*P* = 0.086) were not significantly different between the risk groups. To clarify the interrelation of stWNT5A to the single factors of the PCa risk stratification, GS and TNM‐staging items were considered independently. In patients with Gleason Score (GS) ≤ 6, expression of stWNT5A was significantly higher than in patients with GS = 7 (Fig. 2B, *P* < 0.05) and GS ≥ 8 (Fig. 2B, *P* < 0.01). Regarding local tumor expansion, we compared the expression of stWNT5A in patients with a localized (pT2) to locally advanced tumors (pT3 and pT4) but no significant results revealed (Fig. 2C, *P* = 0.06). In this context, we also analyzed the expression of stWNT5A in each pT‐stage separately. Compared to pT2 patients expression of stWNT5A was significantly lower in pT3 (*P* < 0.05), but not in pT4 patients (data not shown). The absence (pN0) or presence of lymph node metastases (pN1) was not related to stWNT5A expression (Fig. 2D). To determine the relevance in the context of tumor spreading, we analyzed the expression of stWNT5A in pN0 and pN1 patients in relation to the groups GS ≤ 6, GS = 7 and GS ≥ 8. Compared to patients staged as pN0 and GS ≤ 6, expression of stWNT5A was significantly lower in pN1 patients independent of GS (Fig. 2E, *P < 0.05, **P < 0.01, ***P < 0.001). No significant differences were found in patients staged as pN1 and GS ≤ 6 compared to the other groups. Linear regression analysis of PSA serum levels to stWNT5A expression was not significant (Fig. 2F, *P* = 0.093, *r* ^2^ = 0.007). In summary, high stWNT5A went along with non‐high‐risk PCa, low GS, and localized PCa without lymph node metastases. However, multivariate analysis of these clinically relevant parameters revealed no significant results (data not shown).

#### In PCa, stWNT5A correlates negatively to proliferation and apoptosis without impact on WNT5A receptors FZD5 and RYK

**Fig. 3:**
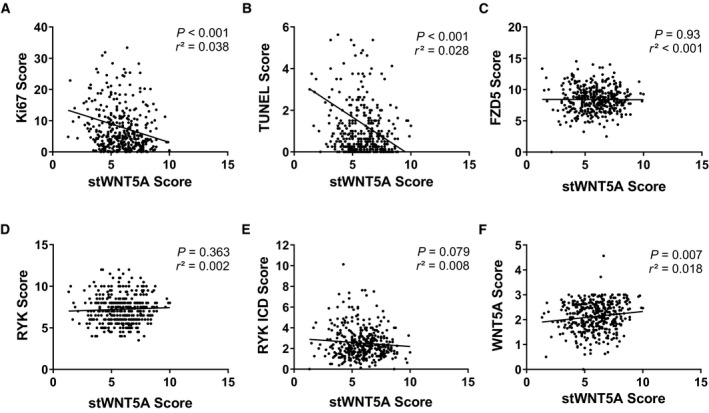
Stromal WNT5A expression is inversely related to proliferation and apoptosis markers in PCa without influence on WNT5A receptors. (A) Linear regression analysis of tumor Ki67 expression to stWNT5A (*P* < 0.001, *r* ^2^ = 0.038). (B) Linear regression analysis of TUNEL staining to stWNT5A (*P* < 0.001, *r* ^2^ = 0.028). (C) Linear regression analysis of FZD5‐Receptor to stWNT5A (*P* = 0.930, *r* ^2^ < 0.001). (D) Linear regression analysis of cytoplasmatic RYK receptor (RYK) to stWNT5A (*P* = 0.363, *r* ^2^ = 0.002). (E) Linear regression analysis of nuclear RYK receptor (RYK ICD, intracellular domain) to stWNT5A (*P* = 0.079, *r* ^2^ = 0.008). (F) Linear regression analysis of WNT5A to stWNT5A expression (*P* < 0.007, *r* ^2^ = 0.018). Correlation was calculated using Pearson *r* correlation and linear regression analysis.

Expression of stWNT5A was further assessed with respect to proliferation and apoptosis markers. Linear regression analysis of stWNT5A and the proliferation marker Kiel‐67 (Ki‐67) revealed an inverse correlation (Fig. 3A, *P* < 0.001, *r* ^2^ = 0.038). Furthermore, stWNT5A expression was inversely associated with TUNEL staining (Fig. 3B, *P* < 0.001, *r* ^2^ = 0.028). Known mediators of WNT5A‐induced effects include the receptors Frizzled 5 (FZD5) and receptor tyrosine kinase (RYK). Linear regression analysis of stWNT5A in relation to FZD5, cytoplasmatic RYK, and nuclear RYK ICD (intracellular domain) has shown no significant interrelation (Fig. 3C–E). To confine the different effects of WNT5A and stWNT5A, a linear regression analysis between stWNT5A and WNT5A was performed, showing a weak, but significant positive correlation of these two parameters (Fig. 3F, *P* = 0.007, *r* ^2^ = 0.018). However, OS (*P* = 0.48) and CSS (*P* = 0.96) of patients with high expression of WNT5A and stWNT5A were not superior to patients with low expression of WNT5A and/or stWNAT5A (data not shown).

## Discussion

Despite the current diagnostic and therapeutic progress, PCa after metastatic spreading into bone confers still a poor prognosis. The microenvironment of the primary tumor cells, which leads to metastatic evasion, is poorly understood. To investigate the local processes in the tumor microenvironment, we focused on the surrounding stroma and expression of stWNT5A in primary PCa. Expression of stWNT5A histologically clearly differed from that of the tumor cells. Tumor‐free tissue and BPH have shown a higher expression of stWNT5A compared to PCa tissue. These findings are supported by *in vivo* experiments in mice, which indicated a suppressive role of stroma‐derived WNT5A (stWNT5A) on proliferative epithelial cells of the prostate gland [27]. This implies a regulatory role of high stWNT5A in limiting epithelial growth. Thus, it appears that high stWNT5A expression is a characteristic feature of benign tissue. In contrast to WNT5A expression in tumor cells, which was associated with a longer OS [26], stWNT5A had no influence on overall or CSS.

To unravel the clinical relevance, we analyzed stWNT5A expression in patients with high‐risk and non‐high‐risk PCa. High‐risk patients showed a significantly lower expression of stWNT5A compared to non‐high‐risk patients. Tumor stage, GS, and PSA are known relevant parameters for PCa patients [30] and part of the risk stratification. To clarify the role of each parameter, we performed single analyses. A high expression of stWNT5A was associated with lower GS and also found in localized PCa without lymph node metastases.

Local extension of PCa might be related to stWNT5A expression as there was a significantly higher expression in pT2 compared to pT3. WNT5A is a chemoattractant for PCa cells [29]. In pT2 tumors, a higher expression in the prostatic gland may keep the cancer locally confined. A lower expression of stWNT5A was associated with an extraprostatic extension (pT3). The further expansion to surrounding organs (pT4) is a change in host tissue for the cancer [33]. This might explain the nonsignificant results between pT2 and pT4 and between localized (pT2) and locally advanced disease (pT3 + pT4). The locally advanced expansion seems no longer be related to the expression patterns of stWNT5A. To clarify this assumption, future investigations are needed.

Patients with GS = 7 and GS ≥ 8 had a lower expression of stWNT5A compared to patients with GS ≤ 6. To clarify the influence of stWNT5A in the context of the potential of the tumor to spread into lymph nodes, a comparison of stWNT5A in the group with the best prognosis and no lymph node metastases (GS < 7, pN0) to the other groups with lymph node metastases (pN1) was performed. Among all three GS subgroups, pN1 scored patients had a lower expression of stWNT5A. These findings may imply a suppressive role of stWNT5A in context of PCa spreading to lymph nodes. WNT5A is known to inhibit the proliferation of lymphatic B‐cells and has a function as a tumor suppressor in hematopoietic tissue [20]. This suppressive effect may exist for PCa tissue, too. This might imply a stWNT5A‐controlled invasion affinity of PCa cells to lymphatic tissue that is unknown, yet. The interrelation between stWNT5A and metastatic dissemination of tumor cells into lymph nodes should be addressed in future studies.

Tumor expansion is commonly associated with a high rate of proliferation and low apoptosis. To clarify the role of stWNT5A on tumor behavior, we correlated it to the expression of proliferation and apoptosis markers. Both decreased with a higher expression of stWNT5A. Of note, unpublished analysis of our TMA revealed a negative correlation of tumor‐derived WNT5A with proliferation (*P* = 0.003, *r* ^2^ = 0.001,) but no correlation with apoptosis. *In vitro* experiments revealed that overexpression of WNT5A in PCa cells leads to a decrease of proliferation and an increase in apoptosis [26]. Our findings suggest that externally derived WNT5A as from stroma tissue assessed here, application of recombinant WNT5A [23] or WNT5A overexpression [26] could all lead to a local deceleration of tumor growth and cell turnover. This hypothesis is supported by similar findings in bone metastases, where recombinant WNT5A was able to induce dormancy in PCa cells in a reversible manner [25]. These findings imply a different influence of tumor‐ and stWNT5A in PCa behavior. Our results are consistent with studies, where WNT5A has tumor‐suppressive effects [16, 17, 18, 19, 20]. In hematopoietic stem cells, Wnt5a was able to inhibit proliferation and apoptosis and promote a retention in G_0_‐state of the mitosis [35]. Otherwise, the ambivalent character of WNT5A and a tumor‐promoting potential [12, 13, 14, 15] is already known, too. In lung cancer, proliferation was positively correlated with WNT5A expression [14].

As stWNT5A correlated positively with tumor‐derived WNT5A, the communication mechanisms of stWNT5A were also addressed in our study. In contrast to our previous findings, where tumor‐derived WNT5A was corresponding with FZD5 and RYK receptor [32] no interrelation of these receptors to stWNT5A was observed in this study. This suggests that stWNT5A may interact in a different way. Experimental blocking of Tyrosine‐protein kinase transmembrane receptor (ROR2) receptors reversed the positive effect of WNT5A in cancer metastases [34] and may induce dormancy in bone in a WNT5A/ROR2‐dependent manner [25]. Presumably, the ROR2 receptor may contribute to the evasion of primary PCa cells.

We recognize potential limitations. In comparison with our previous study [26], the observational period was extended between 2015 and the current analysis from 10 years to a total of 21 years. Therefore, the comparison of stWNT5A‐ to WNT5A‐effects is hindered by the improved therapeutic options for PCa and other diseases resulting in a limited survival analysis. In addition, for some patients, no information was available about the development of bone metastases. Nevertheless, the large number of patients in the TMA provided us with powerful information about the local microenvironment of primary PCa.

In conclusion, stWNT5A appears to play a role in dissemination of PCa to other tissues and acts in a different way than tumor‐derived WNT5A. It may particularly be involved in dissemination of PCa to lymph nodes. As stWNT5A was higher expressed in benign prostate tissue and inversely associated with proliferation and apoptosis in PCa, it might be a possible target for future therapeutic approaches.

## Materials and methods

### Characteristics of the tissue microarray

A TMA was investigated in accordance to the standards set by the Declaration of Helsinki. It consisted of samples obtained from 400 PCa patients, who underwent RP without previous androgen deprivation therapy and control samples from 41 patients with BPH treated at the Department of Urology of the University Hospital Dresden between 1996 and 2005. The study was approved by the Internal Review Board of the Technische Universität Dresden (EK194092004, EK195092004, EK59032007). Every patient agreed to participate with a signed informed consent. Two pathologists (GB, US) reviewed the formalin‐fixed and paraffin‐embedded specimen for the presence of representative acinar adenocarcinomas. Four tumor containing, two tumor‐free tissue cores and one core of BPH control tissue were fixed in a total of fourteen paraffin TMA blocks.

The cohort was analyzed for the relevant clinical parameters [30] serum PSA, GS, and TNM‐stage and divided according to clinical risk stratification guidelines for local recurrence and metastatic spreading of PCa [31] into a non‐high‐risk (≤ pT2 and pN0 and GS ≤ 7) and a high‐risk group (≥ pT3 and/or pN1 and/or GS ≥ 8).

### Immunohistochemical staining

A previously performed immunohistochemical staining of the TMA for WNT5A [26] was analyzed focusing on stWNT5A expression. The TMA blocks were cut in 2‐µm‐thick sections. These sections were dewaxed in xylene and rehydrated in decreasing concentrations of ethanol. Afterward, they were heat‐treated for antigen retrieval. Endogenous peroxidases were blocked in 0.3% water/PBS‐solution for 10 min at room temperature. Nonspecific sites were treated with the blocking buffer of the VECTASTAIN Elite ABC Kit (VECTOR Laboratories, Burlingame, CA, USA) for 45 min at room temperature. The sections were incubated using a 1 : 250 dilution of a monoclonal anti‐WNT5A antibody (Abnova, Heidelberg, Germany) at 4 °C overnight. Afterward, the slides were treated with an anti‐mouse secondary antibody, conjugated to biotin and using avidin conjugated horseradish‐peroxidase with diaminobenzidine as substrate (DAKO, Hamburg, Germany). A pathologist (US) confirmed validity of the staining. Scoring of the expression of WNT5A in the tumor surrounding stroma (stWNT5A) was performed based on staining intensity as 0 (no staining), 1 (weak staining), 2 (moderate staining), or 3 (strong staining) and considering the quantity of the stained stromal area as 0 (0%), 1 (1–25%), 2 (26–50%), 3 (51–75%), or 4 (76–100%). Staining intensity and surface area scores were multiplied to generate an expression score. Two independent scientists (WK, SC) performed the scoring. Divergent scores were reevaluated until consensus was reached. Average values of six malignant and two benign cores were analyzed. To verify whether stWNT5A expression impacts the survival of the patients, we divided the cohort by the median of the stWNT5A score in a high expression and a low expression group.

Receptor tyrosine kinase and FZD5 are known receptors of WNT5A in the noncanonical pathway [32]. Immunohistochemical staining for FZD5 (Abcam, Cambridge, UK, 1 : 100 dilution), cytoplasmatic RYK (RYK), and nuclear RYK receptor (RYK ICD) (Abcam, 1 : 100 dilution) was performed previously and scored similar to WNT5A. Tumor cell proliferation was assessed by Ki‐67 staining (Ventana; Roche Diagnostics GmbH, Mannheim, Germany, 1 : 50 dilution). To investigate the TMAs for apoptosis, a TUNEL staining (TdT‐mediated dUTP‐biotin nick end labeling) was performed (TACS‐XL® Basic Kit; Trevigen, Inc., Gaithersburg, MD, USA).

### Statistical analysis

For statistical analysis, graph pad prism 8 (GraphPad Software, San Diego, CA, USA) was used. Student’s *t*‐test was conducted for single group comparisons. OS and CSS were analyzed using the Kaplan–Meier estimation. For comparison of probability of OS and CSS, Log‐Rank (Mantel‐Cox) test was conducted. Interactions of variables were analyzed by linear regression analysis. Correlation analysis was performed by Pearson’s *r*‐test. Cox‐regression was performed for multivariate analysis. *P*‐values < 0.5 were determined as statistically significant. All data were checked by the Kolmogorov–Smirnov test for normality. If data were not normally distributed, a nonparametric test (Mann–Whitney *U* test) was performed.

## Conflict of interest

The authors declare no conflict of interest.

## Author contributions

WK, SC, MR, LH, and CH conceptualized the data; WK, SC, MR, and US performed the methodology; WK and SC performed software; WK, SC, AB, MR, CH, and LH performed the formal analysis; WK, SC, and US performed investigation; WK wrote original draft preparation; WK, SC, AB, GF, SF, MR, CH, and LH reviewed and edited the manuscript; WK and SC visualized the manuscript; MR, SF, GB, CT, KDS, CH, and LH supervised the manuscript; MR, CH, and LH involved in project administration; MR, CH, and LH involved in funding acquisition. All authors have read and agreed to the published version of the manuscript.

## Data Availability

The data that support the findings of this study are available from the corresponding author (mailto: Lorenz.Hofbauer@uniklinikum-dresden.de) upon reasonable request.
